# Economic analysis of adjuvanted trivalent influenza vaccine for older adults in the Korean National Immunization Program

**DOI:** 10.3389/fpubh.2026.1833971

**Published:** 2026-10-08

**Authors:** Eunha Shim, Byung Wook Eun, Won Suk Choi

**Affiliations:** 1 Department of Mathematics, Soongsil University, Seoul, Republic of Korea; 2 Department of Pediatrics, Nowon Eulji University Hospital, Seoul, Republic of Korea; 3 Division of Infectious Diseases, Department of Internal Medicine, Korea University Ansan Hospital, Korea University College of Medicine, Ansan, Republic of Korea

**Keywords:** dynamic transmission model, economic analysis, older population, influenza vaccination, MF59-adjuvanted vaccine, National Immunization Program, South Korea

## Abstract

**Background:**

Seasonal influenza causes substantial morbidity and mortality among older adults in South Korea despite high vaccination coverage. The MF59-adjuvanted trivalent influenza vaccine (aTIV) has shown superior immunogenicity in older populations, but its economic value within the Korean National Immunization Program (NIP) has not been evaluated using a dynamic transmission model that captures indirect protection. This study evaluated the budget impact of introducing aTIV into the Korean NIP for adults aged ≥65 years.

**Methods:**

We developed an age-structured dynamic compartmental model to simulate seasonal influenza transmission in South Korea, allowing estimation of both direct and indirect protection effects. From the healthcare payer perspective and over a single influenza season, we performed a budget impact analysis complemented by a cost–benefit comparison of avoided medical costs against incremental vaccination expenditures. Two aTIV strategies were compared against standard quadrivalent influenza vaccine (QIV) within the NIP framework: Strategy I (aTIV for adults aged ≥75 years) and Strategy II (aTIV for all adults aged ≥65 years). Deterministic one-way sensitivity analyses examined relative vaccine effectiveness (rVE) of aTIV versus QIV across 5–30% and seasonal attack rate across ±20% of the 8.0% baseline, and a break-even analysis identified the rVE threshold at which incremental costs equal avoided treatment costs. A probabilistic sensitivity analysis was also conducted to quantify joint parameter uncertainty.

**Results:**

At baseline (rVE 25%, attack rate 8.0%), Strategy I required $24.6 million in additional vaccine investment, with 52% offset by treatment savings, yielding a net budget impact of $11.7 million (+0.9% of total influenza-related healthcare expenditure). Strategy II required $54.4 million in additional costs with 57% offset ($31.2 million in savings), resulting in a net impact of $23.2 million (+1.8%). Strategy II prevented 535 deaths and over 15,000 hospitalizations—2.2-fold greater than Strategy I. The incremental cost of aTIV decreased with increasing transmission intensity, approaching cost neutrality during severe epidemic seasons. The break-even rVE at which aTIV becomes cost-saving was 44%, above central meta-analytic estimates (10–25%) but potentially attainable in severe seasons or specific subpopulations.

**Conclusion:**

Introduction of aTIV into the Korean NIP is budget-feasible with substantial health benefits, requiring only a 0.9–1.8% increase in total influenza-related healthcare expenditure. At current rVE estimates (≤30%), aTIV does not achieve cost savings, but the additional fiscal burden remains modest. Strategy II offers superior health outcome value and is preferred when budget permits, while Strategy I provides a fiscally conservative entry point. The value proposition of aTIV is most favorable during severe influenza seasons.

## 1 Introduction

Seasonal influenza remains a significant public health concern globally and in South Korea, particularly among older adults. Influenza infection in older adults is associated with higher rates of complications, hospitalization, and mortality compared with younger age groups, leading to substantial disease burden and healthcare utilization (1). Adults aged ≥65 years are disproportionately affected by influenza-associated morbidity and mortality, accounting for 70–90% of influenza-related deaths (2) and 50–70% of hospitalizations in developed countries (3). In South Korea specifically, the total socioeconomic burden of seasonal influenza has been estimated at $156–316 million annually among adults, with costs rising substantially during severe epidemic seasons, particularly those dominated by A/H3N2 (4). Adults aged ≥65 years bear a disproportionate share of this burden through higher rates of hospitalization, intensive care admission, and mortality (3).

The National Immunization Program (NIP) in South Korea provides free seasonal influenza vaccination to adults aged ≥65 years, achieving one of the highest vaccination coverage levels globally, exceeding 80% in recent seasons (5). However, substantial residual disease burden persists among vaccinated older individuals due to immunosenescence, the age-related decline in immune function with aging (6). These changes translate into lower vaccine effectiveness (VE) in older adults compared with younger populations (7).

The MF59 adjuvant, an oil-in-water emulsion, enhances vaccine immunogenicity by improving antigen uptake and presentation, promoting dendritic cell maturation, and broadening antibody responses against diverse epitopes (8). These mechanisms are well suited to overcoming immunosenescence. A meta-analysis by Nicolay et al. of 23 randomized controlled trials (*n* = 11,105) reported significantly higher seroconversion rates against H3N2 with the MF59-adjuvanted trivalent influenza vaccine (aTIV) compared with non-adjuvanted comparators (percent difference 10.5, 95% CI 6.6–14.5) (9). Real-world effectiveness studies have further demonstrated that aTIV provides superior protection against influenza-related outcomes compared with standard-dose non-adjuvanted vaccines in adults aged ≥65 years, with a relative VE of 16.9% (95% CI: 1.7–29.8) against influenza-related medical encounters (10) and a relative VE of 19.0% (95% CI: 16.3–21.6) against influenza- or pneumonia-related hospitalizations (11).

Several high-income countries have incorporated aTIV or aQIV into their National Immunization Programs or issued preferential recommendations for enhanced vaccines in older populations, including the United States (12), the United Kingdom (13), Italy (14), and Australia (15). International economic assessments have consistently supported the economic value of aTIV, with studies from the USA, UK, Italy, Canada, South Korea, Brazil, and Argentina demonstrating favorable cost-effectiveness profiles (16–22).

To date, two Korean studies have evaluated enhanced influenza vaccines for older adults. Yun et al. used a static Markov model that excluded indirect protection effects and reported cost-utility results without budget impact estimates (21). Song and Shim applied a dynamic model but evaluated aQIV, a distinct vaccine formulation, through cost-effectiveness rather than budget impact analysis (23). Neither study quantified the fiscal impact of aTIV introduction into the Korean NIP nor compared targeted versus universal age-based deployment strategies.

Given Korea’s rapidly aging population, with adults aged ≥65 years having reached 20% in 2025 and projected to exceed 40% by 2050 (24), and the substantial residual disease burden despite high vaccination coverage, the present study evaluated the budget impact of introducing aTIV into the Korean NIP for adults aged ≥65 years, compared with the standard quadrivalent influenza vaccine (QIV), from a healthcare payer perspective. We applied an age-structured dynamic transmission model to capture both direct and indirect protection effects, compared two age-differentiated strategies (aTIV for ≥75 years versus aTIV for ≥65 years), and conducted a break-even analysis to identify the relative vaccine effectiveness threshold at which aTIV becomes cost-saving. This is the first dynamic transmission model-based economic evaluation of aTIV in the Korean NIP.

## 2 Materials and methods

### 2.1 Study design

The influenza transmission model includes the entire Korean population of approximately 51.2 million individuals across 17 age groups to capture transmission dynamics and indirect protection effects. The vaccination policy comparison focuses on adults aged ≥65 years, who comprise approximately 10.3 million individuals (20% of the total Korean population) and are the eligible target population under the current NIP (24). The older population can be are further divided into three subgroups (65–69, 70–74, and ≥75 years) to account for age-dependent variation in influenza attack rates, vaccine effectiveness, healthcare utilization patterns, and vaccination coverage rates (2, 3, 25).

The analysis employs a single influenza season time horizon, aligning with NIP vaccine procurement decision cycles and avoiding compounding uncertainty from multi-season assumptions regarding year-to-year variation in epidemic severity, circulating strains, and vaccine-virus match (26). With a time horizon of less than 1 year, discounting of future costs is not required (26).

Three vaccination strategies were compared (Table 1). The comparator strategy applied QIV to all vaccinated individuals (standard of care). Strategy I provided aTIV to adults aged ≥75 years only, with QIV maintained for those <75 years. Strategy II provided aTIV to all adults aged ≥65 years, with QIV maintained for those <65 years. The selection of these comparators reflects realistic policy options under consideration, with Strategy I acknowledging potential budget constraints and the observation that influenza-related hospitalization and mortality increase substantially after age 75 (3, 27).

**Table 1:** Seasonal influenza vaccination strategies.

Strategy	<65 years	65–74 years	≥75 years	Description
Comparator	QIV	QIV	QIV	Standard of care
Strategy I	QIV	QIV	aTIV	Targeted aTIV for ≥75 years
Strategy II	QIV	aTIV	aTIV	Universal aTIV for ≥65 years

aTIV, adjuvanted trivalent influenza vaccine; QIV, quadrivalent influenza vaccine.

### 2.2 Epidemiological model

We constructed an age-structured dynamic transmission model extending the susceptible-exposed-vaccinated-infected-recovered (SEVIR) framework to compare the economic impact of aTIV introduction (28, 29). The model stratifies the population into 17 age groups: 0–6 months, 7 months–4 years, 5–9 years, 10–14 years, and subsequent 5-year intervals up to 70–74 years, with a final group of ≥75 years. Individuals transition through mutually exclusive health states based on their infection and care-seeking status, including susceptible ( $S_{k}$ ), vaccinated with QIV ( $V_{k}$ ) or aTIV ( $W_{k}$ ), exposed ( $E_{k}$ ), asymptomatically infected ( $A_{k}$ ), symptomatically infected ( $I_{k}$ ), receiving outpatient care without ( $O_{k}$ ) or with ( $X_{k}$ ) complications , hospitalized without ( $H_{k}$ ) or with ( $Y_{k}$ ) complications, recovered ( $R_{k}$ ), and deceased ( $D_{k}$ ). The subscript k denotes the age group ( k=1,2,…,17 ). At any time t , the total population in age group k is given by *N_k_(t)* = *S_k_(t)* + *V_k_(t)* + *W_k_(t)* + *E_k_(t)* + *A_k_(t)* + *I_k_(t)* + *O_k_(t)* + *H_k_(t)* + *X_k_(t)* + *N_k_(t)* + *Y_k_(t)* + *R_k_(t)* (see Figure 1).

**Figure 1:**
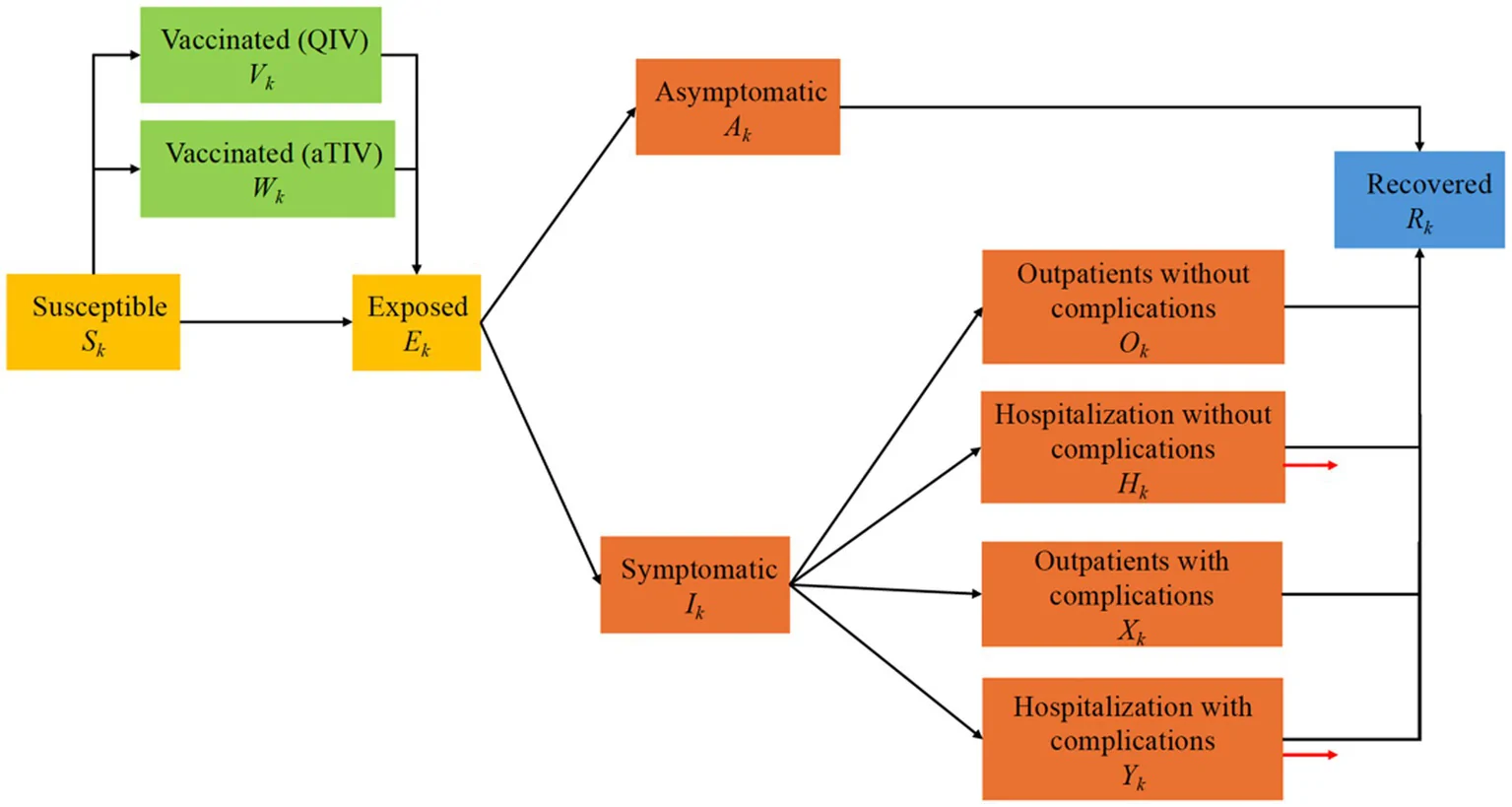
Model diagram of influenza transmission and vaccination. Red arrows indicate influenza-related deaths.

Age-specific vaccination rates for QIV ( $\phi_{k}(t)$ ) and aTIV ( $\psi_{k}(t)$ ) were parameterized to reflect monthly uptake patterns observed across age groups in South Korea (30). Vaccine-induced protection is characterized by efficacy parameters $\delta_{V,k}$ for QIV and $\delta_{W}$ for aTIV. In accordance with national immunization guidelines that recommend vaccination from 6 months of age, $\phi_{1}(t)=0$ and $\psi_{1}(t)=0$ for infants aged 0–5 months.

The force of infection acting on susceptible individuals in age group k , denoted $\lambda_{k}(t)$ , incorporates a composite parameter $u_{k}$ that combines the per-contact transmission probability and the age-specific susceptibility for age group *k* (calibrated to age-stratified case data), inter-group contact patterns ( $c_{\mathrm{kj}}$ ), and differential infectiousness across disease states. Asymptomatic cases and outpatients are assumed to have reduced transmissibility relative to symptomatic individuals not yet seeking care, captured by scaling factors $\omega_{A}$ and $\omega_{O}$ , respectively. The force of infection is expressed as $\lambda_{k}(t)=u_{k}\sum_{j=1}^{17}c_{\mathrm{kj}}\frac{I_{j}+\omega_{A}A_{j}+\omega_{O}O_{j}+\omega_{O}X_{j}}{N_{j}-H_{j}-Y_{j}-D_{j}}$ . Hospitalized individuals ( $H_{k}$ and $Y_{k}$ ) are excluded from the transmission process, reflecting their isolation from the general population.

Disease progression is governed by several rate and probability parameters. The average duration of the latent period and the reporting delay are represented by 1/ ρ and 1/ $\sigma_{D}$ , respectively. The probability of developing symptoms following infection is α , and the age-specific probabilities of complications given symptomatic infection are $\tau_{k}$ . Among symptomatic cases, age-specific probabilities of outpatient visits without complications ( $\eta_{k}$ ) and with complication ( $\varepsilon_{k}$ ) were taken from the National Health Insurance claims data from South Korea spanning 2014–2019 (31). Recovery rates also vary across disease states. The recovery rate is $\gamma_{A}$ for asymptomatic cases that recover without seeking hospital care, $\gamma_{O}$ for outpatients, and the age-specific rates ( $\gamma_{H,k}$ ) for hospitalized patients. Hospitalized patients can also experience influenza-attributable mortality at rate $\mu_{k}$ .

Given the short time horizon of the analysis, demographic processes including births, background mortality, and aging were omitted. The system dynamics are governed by the following ordinary differential equations:

$$\frac{dS_{k}(t)}{\mathrm{dt}}=-(\phi_{k}(t)+\psi_{k}(t))S_{k}(t)-\lambda_{k}(t)S_{k}(t),$$

$$\frac{dV_{k}(t)}{\mathrm{dt}}=\phi_{k}(t)S_{k}(t)-(1-\delta_{V,k})\lambda_{k}(t)V_{k}(t),$$

$$\frac{dW_{k}(t)}{\mathrm{dt}}=\psi_{k}(t)S_{k}(t)-(1-\delta_{W})\lambda_{k}(t)W_{k}(t),$$

$$\begin{array}{l} \frac{dE_{k}(t)}{\mathrm{dt}}=\lambda_{k}(t)S_{k}(t)+(1-\delta_{V,k})\lambda_{k}(t)V_{k}(t)\\ +(1-\delta_{W})\lambda_{k}(t)W_{k}(t)-\rho E_{k}(t), \end{array}$$

$$\frac{dA_{k}(t)}{\mathrm{dt}}=(1-\alpha )\rho E_{k}(t)-\gamma_{A}A_{k}(t),$$

$$\frac{dI_{k}(t)}{\mathrm{dt}}=\alpha \rho E_{k}(t)-\sigma_{D}I_{k}(t),$$

$$\frac{dO_{k}(t)}{\mathrm{dt}}=(1-\tau_{k})\eta_{k}\sigma_{D}I_{k}(t)-\gamma_{O}O_{k}(t),$$

$$\frac{dH_{k}(t)}{\mathrm{dt}}=(1-\tau_{k})(1-\eta_{k})\sigma_{D}I_{k}(t)-(\gamma_{H,k}+\mu_{k})H_{k}(t),$$

$$\frac{dX_{k}(t)}{\mathrm{dt}}=\tau_{k}\varepsilon_{k}\sigma_{D}I_{k}(t)-\gamma_{O}X_{k}(t),$$

$$\frac{dY_{k}(t)}{\mathrm{dt}}=\tau_{k}(1-\varepsilon_{k})\sigma_{D}I_{k}(t)-\gamma_{H,k}Y_{k}(t),$$

$$\frac{dR_{k}(t)}{\mathrm{dt}}=\gamma_{A}A_{k}(t)+\gamma_{O}(O_{k}(t)+X_{k}(t))+\gamma_{H,k}(H_{k}(t)+Y_{k}(t))$$

At *t* = 0, corresponding to the start of the influenza season, a base seed of 0.06% of each age group’s population was distributed across the infection compartments, with one half in the exposed compartment and the remaining half split in a 1:2 ratio between the asymptomatic and symptomatic compartments. The remaining ~99.94% assigned to the susceptible compartment and all other compartments initialized to zero. Inter-group contact patterns were derived from the synthetic age-structured contact matrix for South Korea by Prem et al. (32), as nationally representative empirical contact data for the Korean population are not available. This matrix was constructed using a Bayesian hierarchical model fitted to POLYMOD survey data and projected to South Korea using country-specific demographic structure, household composition, school enrolment, and labor-force participation data. Asymptomatic cases and outpatients are assumed to have 50% reduced transmissibility relative to symptomatic individuals not yet seeking care (33–35). Age-specific probabilities of outpatient visits and hospitalization were estimated from National Health Insurance claims data from South Korea spanning 2014–2019 (31). Given the short time horizon, demographic processes including births, background mortality, and aging were omitted. All model simulations, calibration, and economic analyses were implemented in MATLAB (version R2023a, MathWorks, Natick, MA, USA). The system of ordinary differential equations was solved numerically using the ode45 solver, and parameter calibration was performed with the *lsqnonlin* nonlinear least-squares routine.

### 2.3 Epidemiological inputs

Epidemiological inputs were derived from national surveillance data and published literature (Table 2). Overall vaccination coverage in the base-case scenario was assumed to be 48%, consistent with the observed uptake rate during the 2023–2024 influenza season in South Korea (36). For adults aged ≥65 years, who represent the primary target population under the current NIP, an age-specific coverage of 78% was applied based on national surveillance estimates. The temporal distribution of vaccine administration was parameterized to reflect seasonal uptake patterns observed in routine immunization practice (30).

**Table 2:** Description of epidemiological parameters and baseline values.

Age (years)															
0–4	5–9	10–14	15–19	20–24	25–29	30–34	35–39	40–44	45–49	50–54	55–59	60–64	65–69	70–74	75+
Population (50)															
1,271,776	1,868,342	2,324,303	2,294,962	2,647,814	3,307,842	3,479,212	3,145,351	3,882,799	3,840,024	4,433,119	4,273,251	4,191,644	3,627,139	2,399,923	4,229,720
Vaccine efficacy (43, 44, 51)															
QIV VE, $\delta_{V,k}$															
0.46	0.46	0.46	0.46	0.46	0.46	0.46	0.46	0.46	0.46	0.46	0.46	0.46	0.45	0.45	0.45
rVE aTIV vs. QIV															
													0.25		
Vaccine coverage level (%) (36)															
63.1	80.6	69.1	63.1	30.7	21.4	32.2	32.2	33.8	33.8	32.8	32.8	65.0	65.0	86.7	86.7
Probability of complications given symptomatic infection, $\tau_{k}$ (37)															
0.130	0.828	0.054	0.035	0.024	0.024	0.024	0.024	0.024	0.024	0.041	0.041	0.064	0.122	0.122	0.122
Proportion of outpatients given symptomatic infection without complications, $\eta_{k}$ (37)															
0.824	0.904	0.932	0.955	0.980	0.980	0.980	0.980	0.980	0.980	0.950	0.950	0.920	0.891	0.891	0.891
Proportion of outpatients given symptomatic infection with complications, $\varepsilon_{k}$ (37)															
0.824	0.904	0.932	0.955	0.980	0.980	0.980	0.980	0.980	0.980	0.950	0.950	0.920	0.549	0.549	0.549
Probability of manifesting symptoms, α (52)															
0.670															
Reporting delay ( day) , $1/\sigma_{D}\;$ (53)															
1															
Relative infectiousness of outpatients, $\omega_{O}$ (33)															
0.5															
Duration of latent period ( day),1/ρ (54)															
1.2															
Recovery rate of outpatients $\left(\mathrm{da}y^{-1}\right)$ , $\gamma_{O}$ (54)															
0.333															
Recovery rate of infected individuals without symptoms $(\mathrm{da}y^{-1}),\gamma_{A}\;$ (54)															
0.250															
Recovery rate of hospitalization $\left(\mathrm{da}y^{-1}\right)$ , $\gamma_{H,k}\;$ (38)															
0.193	0.215	0.215	0.215	0.218	0.218	0.218	0.218	0.218	0.218	0.206	0.206	0.206	0.180	0.180	0.180
Deaths per 1,000,000 symptomatic cases (37, 55)															
0.560	0.110	0.460	1.24	1.96	1.96	1.96	1.96	1.96	1.96	232	232	232	5,740	5,740	5,740
Probability that an infectious contact leads to infection, $u_{k}$ (calibrated)															
0.249	0.249	0.033	0.033	0.022	0.022	0.022	0.022	0.022	0.022	0.020	0.020	0.020	0.027	0.040	0.055

The model was calibrated to reproduce an annual symptomatic attack rate of 8.0% (37). Age-stratified infection prevalence was derived from influenza case data in the National Health Insurance Service–National Sample Cohort (NHIS–NSC) for the pre-pandemic period (2018–2019), supplemented by influenza laboratory surveillance records (37). Both influenza and influenza-like illness (ILI) cases were included in the calibration dataset to ensure representativeness. Age-specific susceptibility parameters ( $u_{k}$ ) were calibrated using nonlinear least-squares optimization (MATLAB lsqnonlin) to fit simulated case distributions to the empirically observed age structure of influenza infections from NHIS–NSC (37).

Clinical progression parameters, including the probability of developing complications given symptomatic infection, outpatient visit rates, hospitalization rates, and case fatality rates, were stratified by age group and extracted from the NHIS–NSC database (Table 2) (37). Recovery rates for hospitalized patients were derived from length-of-stay data stratified by age (38).

### 2.4 Vaccine effectiveness

In the absence of published data on the absolute efficacy of aTIV against unvaccinated individuals, vaccine effectiveness for aTIV was estimated indirectly using a relative vaccine effectiveness (rVE) approach. rVE is defined as the proportional reduction in residual risk that aTIV provides beyond what QIV already confers, expressed as

$$\mathrm{rVE}=1-(1-{\mathrm{VE}}_{\text{aTIV}})\times (1-{\mathrm{VE}}_{\text{aTIV}}).$$

A value of rVE = 25% therefore means that aTIV reduces the residual risk among QIV recipients by 25%. Based on real-world studies and meta-analyses comparing aTIV with QIV in adults ≥65 years (10, 11, 39), we applied a base-case rVE of 25%. The absolute vaccine effectiveness of aTIV was then derived as

$${\mathrm{VE}}_{\text{aTIV}}=1-(1-{\mathrm{VE}}_{\mathrm{QIV}})\times (1-\mathrm{rVE})$$

Given the inherent uncertainty in rVE estimates, we conducted deterministic sensitivity analyses across rVE values of 5, 10, 15, 20, and 30% to evaluate the robustness of our findings to this key parameter.

### 2.5 Cost inputs

Cost inputs were obtained from the NHIS–NSC database and official NIP procurement records (Table 3). All costs were expressed in 2026 US dollars, adjusted using the consumer price index where applicable (21, 37). The unit cost of SD-QIV was set at $8.46, reflecting the current NIP procurement price (21, 23, 37). A vaccine administration fee of $15.74 per dose was applied (21, 37). As aTIV is not currently included in the Korean NIP, domestic pricing data are unavailable. Based on international pricing comparisons, we assumed that aTIV is priced approximately 1.8-fold higher than QIV (40). Applying this multiplier, the estimated cost of aTIV was $15.22 per dose. Medical treatment costs were stratified by clinical severity (uncomplicated vs. complicated) and care setting (outpatient vs. inpatient). Age-specific cost estimates were extracted from claims data in the NHIS–NSC database (37). Costs for antiviral agents and rapid diagnostic testing were included as components of direct medical expenditures.

**Table 3:** Input data for cost used in the economic analysis (21, 23, 37).

Age	0–4	5–9	10–14	15–19	20–24	25–29	30–34	35–39	40–44	45–49	50–54	55–59	60–64	65–69	70–74	75+
Cost (USD)																
QIV per dose																
	8.46															
aTIV per dose																
	–													15.22		
Vaccine administration																
	15.74															
Uncomplicated outpatient																
	81.08	77.25	74.37	69.49	64.11	64.11	64.11	64.11	64.11	64.11	64.93	64.93	66.10	66.26	66.26	66.26
Complicated outpatient																
	141.28	133.03	128.91	128.59	130.15	130.15	130.15	130.15	130.15	130.15	131.32	131.32	124.99	118.92	118.92	118.92
Uncomplicated hospitalization																
	577.71	611.46	637.75	684.56	736.91	736.91	736.91	736.91	736.91	736.91	971.81	971.81	1130.29	1120.26	1120.26	1120.26
Complicated hospitalization																
	931.37	863.51	778.61	856.53	1045.85	1045.85	1045.85	1045.85	1045.85	1045.85	1052.25	1052.25	1812.86	1318.08	1318.08	1318.08
Anti-influenza agent																
	8.89	14.54	16.05	16.11	16.12	16.12	16.12	16.12	16.12	16.12	16.12	16.12	16.12	16.12	16.12	16.12
Rapid antigen test																
	27.16															

### 2.6 Economic framework: budget impact analysis from the healthcare payer perspective

This analysis quantifies (i) the net fiscal impact of aTIV introduction relative to the current QIV-only NIP (budget impact), and (ii) the balance between avoided treatment costs and incremental vaccination expenditures (cost–benefit). The term ‘net benefit’ here refers to the monetary difference between avoided direct medical costs and incremental vaccination program costs, and is distinct from the incremental net monetary benefit (INMB) of a cost-utility analysis. In our analysis, net benefit is presented but no cost-effectiveness threshold (e.g., willingness-to-pay per QALY) is applied.

The healthcare payer perspective includes only direct medical expenditures and excludes non-medical costs and productivity losses, providing conservative estimates to support vaccine financing decisions. The benefits of aTIV introduction were quantified as avoided medical costs from reductions in influenza infections and severe clinical outcomes, including reductions in outpatient visits, hospitalizations, and influenza-related treatments. Incremental costs included differences in vaccine acquisition costs and downstream medical expenditures associated with each strategy. Net benefit (NB) is calculated as:

$$\begin{array}{l} \mathrm{Net}\;\text{Benefit}=\text{Avoided Direct Medical Costs}\\ -\text{Incremental Vaccination Program Costs} \end{array}$$

A positive net benefit indicates that aTIV adoption generates net savings relative to the QIV-only comparator, while a negative net benefit indicates that incremental vaccination costs exceed treatment cost offsets. This framework enables policymakers to quantify the fiscal consequences of investment in aTIV relative to the existing QIV program, assuming complete replacement of QIV with aTIV in the target age groups.

### 2.7 Sensitivity analyses

The robustness of our findings was evaluated through deterministic one-way sensitivity analyses on two key parameters subject to uncertainty, namely (i) relative vaccine effectiveness (rVE) of aTIV compared with QIV, and (ii) transmission intensity as reflected by the symptomatic attack rate.

For rVE, sensitivity analyses were conducted across a range of 5 to 30%, encompassing the uncertainty bounds reported in published meta-analyses of adjuvanted influenza vaccine effectiveness (39, 41, 42). For attack rate, we varied the base-case value of 8.0% by ±20% (range: 6.4–9.6%) to reflect inter-seasonal variability in influenza transmission intensity. For each parameter combination, we re-estimated health outcomes (symptomatic infections, outpatient visits, hospitalizations, and deaths averted). A break-even analysis was also conducted to identify the rVE threshold at which the net incremental cost of Strategy II equals zero.

In addition to deterministic analyses, we conducted a probabilistic sensitivity analysis (PSA) with 1,000 Monte Carlo iterations to quantify the joint uncertainty of key parameters. In each iteration, four parameter sets were independently sampled. The rVE was drawn from a beta distribution (mean 0.25, 95% CI 0.10–0.40), with bounds derived from published real-world studies and meta-analyses comparing aTIV with QIV (10, 11, 39). The age-specific QIV vaccine effectiveness parameter $\delta_{V,k}$ was sampled by multiplying its base-case value (Table 2) by a random scaling factor from a beta distribution (mean 1.0, 95% CI 0.65–1.30), yielding $\delta_{V,k}$ values approximately 30–60% across age groups and iterations, consistent with strain- and season-specific variability reported in influenza VE meta-analyses (25, 43, 44). The six treatment unit cost components were sampled from gamma distributions with standard deviations set to 20% of the base-case mean values, based on variability observed in NHIS-NSC claims data (37). The aTIV price multiplier was drawn from a gamma distribution with a mean of 1.8, reflecting international pricing comparisons, and a standard deviation of 0.18 (21, 22). For each iteration our ODE model was solved under the comparator and both intervention strategies, and outputs were recorded. We report the median and 2.5th–97.5th percentile range (95% credible interval, CrI) for averted health outcomes and net incremental cost in Table 4.

**Table 4:** Health outcomes and overall costs (USD, $) for each seasonal influenza vaccination strategy.

Outcome	Vaccination strategy		
	All receive QIV	Older adults receive aTIV	
		Individuals aged ≥ 75 receive aTIV	Individuals aged ≥ 65 receive aTIV
Population (65+ years)			
	51,217,221 (10,256,782)		
Coverage			
Number of vaccinated individuals (65+ years)	24,829,077 (8,043,609)		
Influenza outcomes			
Symptomatic infections for all ages (65+ years)	4,054,841 (559,982)	4,005,562 (518,486)	3,929,601 (467,295)
Averted symptomatic infections (all ages), median (95% CrI)	–	46,524 (14,129–112,580)	118,408 (36,555–278,430)
Uncomplicated outpatients for all ages (65+ years)	3,580,975 (440,179)	3,541,460 (407,719)	3,479,001 (367,676)
Complicated outpatients for all ages (65+ years)	194,972 (37,652)	191,874 (34,876)	187,413 (31,451)
Averted outpatients (all ages), median (95% CrI)	–	40,238 (12,241–97,043)	103,500 (32,056–242,426)
Uncomplicated hospitalizations for all ages (65+ years)	245,093 (53,849)	240,744 (49,878)	234,620 (44,979)
Complicated hospitalizations for all ages (65+ years)	47,160 (30,994)	44,847 (28,708)	41,937 (25,889)
Averted hospitalizations (all ages), median (95% CrI)	–	6,283 (1,887–15,554)	14,806 (4,496–36,043)
Averted hospitalizations (65+), median (95% CrI)	–	5,933 (1,745–14,859)	13,215 (3,894–33,076)
Deaths for all ages (65+ years)	3,420 (3,214)	3,181 (2,976)	2,885 (2,682)
Averted deaths (all ages), median (95% CrI)	–	226 (67–567)	506 (149–1,264)
Averted deaths (65+), median (95% CrI)	–	226 (66–566)	503 (148–1,259)
Cost (USD)			
Vaccination program	600,861,677	625,453,086	655,277,630
Treatment-disease	699,199,020	686,285,564	668,031,826
*Total*	1,300,060,697	1,311,738,650	1,323,309,457
*Net incremental cost, median (95% CrI)*	–	11.5 M (−10.2 M to 28.0 M)	23.1 M (−27.2 M to 61.0 M)

Point estimates are from the base-case deterministic analysis. Medians and 95% credible intervals (CrI) for averted outcomes and net incremental cost are from a probabilistic sensitivity analysis with 1,000 Monte Carlo iterations. Averted outpatients and hospitalizations are the sum of uncomplicated and complicated cases averted relative to the comparator.

## 3 Results

### 3.1 Baseline disease burden under current NIP

Assuming QIV provision to all vaccinated individuals (comparator strategy), the model estimated substantial influenza-related disease burden during a single season (Table 4). A total of 4.05 million symptomatic infections were projected across all age groups, with approximately 560,000 cases (13.8%) occurring among adults aged 65 years and older. The healthcare system burden included 3.58 million uncomplicated outpatient visits, 195,000 complicated outpatient visits, and nearly 292,253 hospitalizations (245,093 uncomplicated and 47,160 complicated cases). Influenza-associated mortality was estimated at 3,420 deaths.

Although adults aged ≥65 years represented only 20% of the total Korean population, they accounted for 29.0% of all influenza-related hospitalizations and 94.0% (3,214) of influenza-associated deaths. The case-hospitalization rate among older adults was 15.2% compared with 5.9% in those aged <65 years, and the case-fatality rate was 0.57% versus 0.0059%, representing a 97-fold difference in mortality risk.

### 3.2 Infections averted

Under Strategy I (aTIV for adults aged ≥75 years), the model projected 49,279 fewer symptomatic infections across all age groups, including 41,496 fewer cases among adults aged ≥65 years. This corresponds to a 7.4% reduction in infections among older adults, with the remaining 7,783 averted infections (15.8%) occurring in younger age groups (aged <65 years) through indirect protection. This finding demonstrates that vaccination of older adults generates spillover benefits to the broader community through reduced transmission.

Strategy II (aTIV for all adults aged ≥65 years) prevented 125,240 symptomatic infections overall, a 2.5-fold increase compared with Strategy I. Among adults aged ≥65 years, Strategy II prevented 92,687 infections, a 16.6% reduction in incidence in this population. The indirect protection extended to younger populations was also enhanced, with 32,553 infections averted among individuals under 65 years, a 4.2-fold increase compared with Strategy I. The proportion of averted infections occurring through indirect effects increased from 15.8% under Strategy I to 26.0% under Strategy II.

### 3.3 Hospitalizations averted

Influenza-related hospitalizations, which impose substantial clinical and economic burden, were substantially reduced under both strategies. Strategy I prevented approximately 6,662 hospitalizations, comprising 4,349 uncomplicated and 2,313 complicated cases. Strategy II averted 15,696 hospitalizations (10,473 uncomplicated and 5,223 complicated cases), a 2.4-fold increase compared with Strategy I.

In relative terms, Strategy I reduced total hospitalizations by 2.3% and Strategy II by 5.4%. Among older adults (aged ≥65 years) specifically, Strategy I reduced hospitalizations by 7.4% (from 84,843 to 78,586 cases), while Strategy II achieved a 16.5% reduction (to 70,868 cases). The reduction in complicated hospitalizations, which carry higher costs and greater clinical severity, was proportionally greater, with Strategy I preventing 4.9% and Strategy II preventing 11.1% of complicated hospitalizations. This is relevant from both clinical and economic perspectives, as complicated hospitalizations represent the most resource-intensive component of influenza-related healthcare utilization.

### 3.4 Deaths prevented

Both strategies using aTIV substantially reduced influenza-associated mortality. Strategy I prevented 239 deaths, with 238 (99.6%) occurring among adults aged ≥65 years. Strategy II more than doubled this benefit, averting 535 deaths (532 among older adults). Given the heavy concentration of influenza-related mortality in older adults, these reductions represent a clinically meaningful improvement in outcomes for the most vulnerable individuals.

Strategy I reduced influenza-associated mortality by 7.0% (from 3,420 to 3,181 deaths), while Strategy II achieved a 15.6% reduction (to 2,885 deaths). Among adults aged ≥65 years, Strategy I reduced deaths by 7.4% and Strategy II by 16.6%. The number needed to vaccinate (NNV) with aTIV to prevent one death was 15,230 for Strategy I (3.64 million aTIV doses administered) and 15,028 for Strategy II (8.04 million aTIV doses administered). Notably, Strategy II demonstrates a slightly lower NNV despite including younger adults (65–74 years) with lower baseline mortality risk, attributable to the enhanced indirect protection achieved through broader population coverage and reduced community transmission. Combined with the 2.2-fold greater absolute reduction in deaths, this efficiency advantage further supports Strategy II as the preferred approach when budget constraints permit.

### 3.5 Economic outcomes

Under the comparator strategy (QIV for all vaccinated individuals), total direct medical costs amounted to approximately $1.3 billion, comprising $601 million for the vaccination program and $699 million for influenza-related treatment and hospitalization (Table 5). Treatment costs were distributed across outpatient care ($268.2 million, 38.4%), hospitalization ($258.4 million, 37.0%), anti-influenza agents ($62.1 million, 8.9%), and rapid antigen testing ($110.5 million, 15.8%).

**Table 5:** Detailed cost breakdown by influenza vaccination strategy (USD, millions).

Cost component	QIV (baseline)	Strategy I	Strategy II	Incremental (I)	Incremental (II)
Vaccination program costs	600.9	625.5	655.3	+24.6	+54.4
Treatment costs					
–Outpatient (uncomplicated)	242.8	240.2	236.0	−2.6	−6.8
–Outpatient (complicated)	25.4	25.0	24.5	−0.4	−0.9
–Hospitalization (uncomplicated)	201.4	196.7	190.2	−4.8	−11.2
–Hospitalization (complicated)	57.0	53.9	50.1	−3.0	−6.9
–Anti-influenza agents	62.1	61.3	60.1	−0.8	−2.0
–Rapid antigen test	110.5	109.2	107.1	−1.3	−3.4
Subtotal (treatment)	699.2	686.3	668.0	−12.9	−31.2
Total costs	1,300.1	1,311.7	1,323.3	+11.7	+23.2

Strategy I (aTIV for adults aged ≥75 years) required an additional investment of approximately $24.6 million for vaccine procurement, reflecting the per-dose premium of $6.76 between aTIV ($15.22) and QIV ($8.46) for approximately 3.64 million doses administered to the ≥75 years population. Treatment cost savings amounted to $12.9 million, derived from reductions in uncomplicated outpatient visits ($2.6 million), complicated outpatient visits ($0.4 million), uncomplicated hospitalizations ($4.8 million), complicated hospitalizations ($3.0 million), anti-influenza agents ($0.8 million), and rapid antigen testing ($1.3 million). The net incremental cost was $11.7 million, with a cost offset ratio of 52% (Table 6).

**Table 6:** Cost–benefit analysis summary (USD).

Metric	Strategy I	Strategy II
Avoided treatment costs (benefit)	$12.9 million	$31.2 million
Incremental vaccination costs (cost)	$24.6 million	$54.4 million
Net incremental cost	$11.7 million	$23.2 million

Strategy II (aTIV for all adults aged ≥65 years) increased vaccination program costs by approximately $54.4 million, applied to all 8.04 million vaccinated adults aged ≥65 years. This investment was partially offset by $31.2 million in avoided treatment costs. Specifically, savings included $6.8 million from uncomplicated outpatient visits, $0.9 million from complicated outpatient visits, $11.2 million from uncomplicated hospitalizations, $6.9 million from complicated hospitalizations, $2.0 million from anti-influenza agents, and $3.4 million from rapid antigen testing. The cost offset ratio was 57.4%, higher than Strategy I, reflecting the enhanced indirect protection and greater reduction in transmission intensity achieved through broader population coverage. The net incremental cost was $23.2 million.

From a national budget planning perspective, implementation of Strategy I would require a 4.1% increase in the vaccination program budget, while Strategy II would necessitate a 9.1% increase (Table 7). When treatment cost offsets are accounted for, the net impact on total healthcare expenditure is limited to 0.9% for Strategy I and 1.8% for Strategy II. These modest budget increases translate to substantial health gains, with the incremental cost representing approximately $1.14 per Korean adult aged 65 years and older for Strategy I and $2.26 for Strategy II annually. Although both strategies incur a positive net incremental cost at current aTIV prices, the benefit–cost ratio of 0.57 for Strategy II exceeds that of Strategy I (0.52), indicating that broader coverage generates proportionally greater returns on investment.

**Table 7:** Budget impact summary (USD, millions).

Budget category	QIV (baseline)	Strategy I	Strategy II
Vaccination budget required	600.9	625.5	655.3
Expected treatment expenditure	699.2	686.3	668.0
Total healthcare expenditure	1,300.1	1,311.7	1,323.3
Incremental vaccination budget	—	+24.6 (+4.1%)	+54.4 (+9.1%)
Incremental total budget	—	+11.7 (+0.9%)	+23.2 (+1.8%)

### 3.6 Sensitivity analysis

The absolute vaccine effectiveness (VE) of aTIV depends on both the baseline QIV effectiveness and the rVE. At the baseline rVE of 25%, the absolute VE of aTIV was estimated at 58.9% for adults aged ≥65 years, compared with 45.2% for QIV, a 13.7 percentage point improvement in protection. Across the examined range, absolute VE varied from 47.9% at rVE = 5 to 72.6% at rVE = 50% (Figure 2). This linear relationship indicates that modest improvements in relative effectiveness translate to clinically meaningful gains in absolute protection.

**Figure 2:**
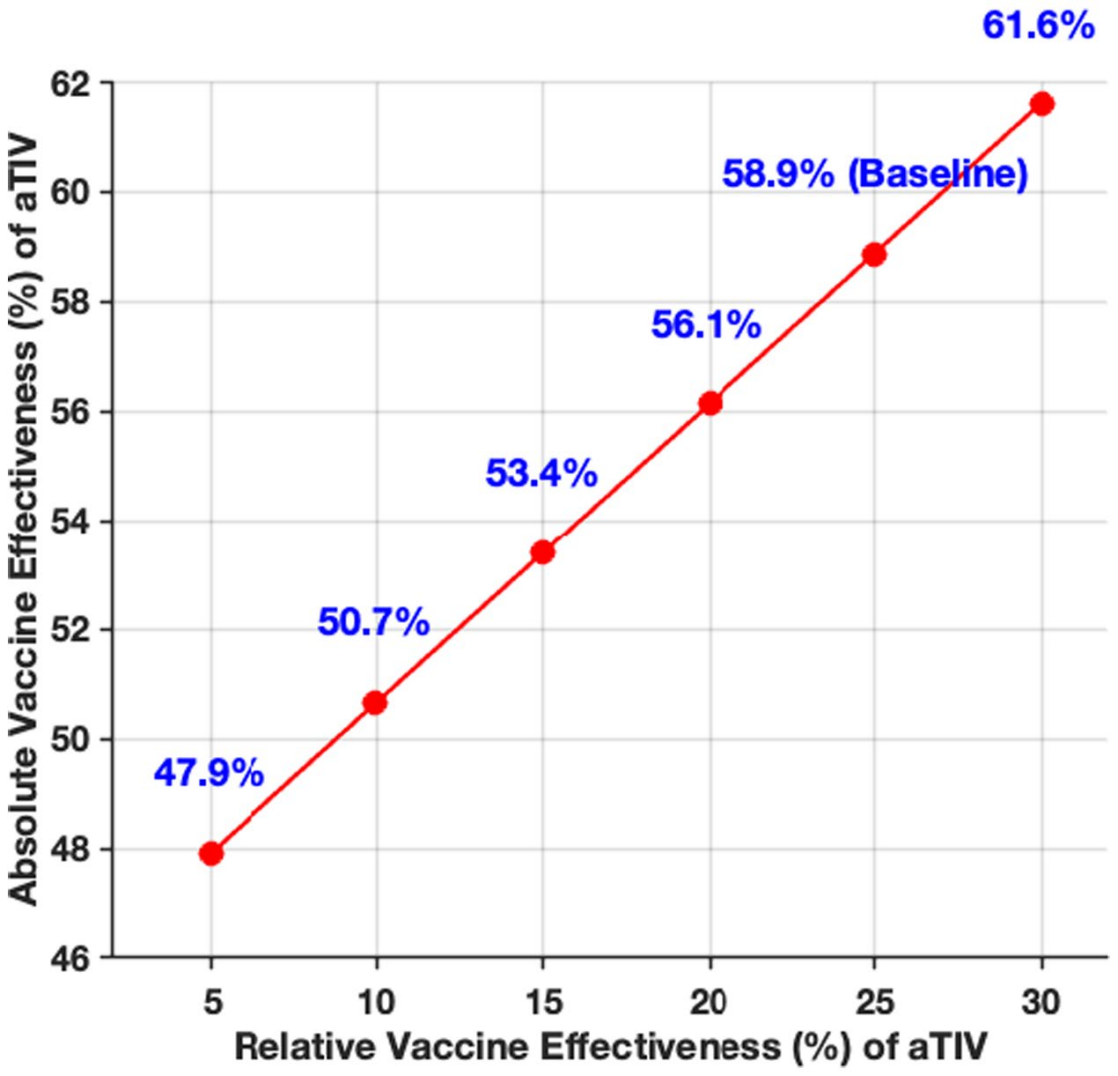
Absolute vaccine effectiveness of aTIV by relative vaccine effectiveness (rVE) for adults aged ≥65 years.

We evaluated how variations in rVE (5 to 30%) affected the number of averted symptomatic infections, outpatients, hospitalizations, and deaths under Strategy II. To capture uncertainty in seasonal transmission dynamics, we conducted analyses under three transmission intensity scenarios: low attack rates (6.4% of population symptomatically infected), baseline (8.0%), and high (9.6%) attack rates.

Under baseline transmission, averted symptomatic infections ranged from approximately 25,300 at rVE = 5% to 150,000 at rVE = 30%, with the baseline estimate of 125,240 at rVE = 25% (Figure 3). Averted outpatients followed a similar pattern, ranging from approximately 22,100 to 131,100. Averted hospitalizations ranged from approximately 3,200 at rVE = 5% to nearly 18,800 at rVE = 30%, and averted deaths ranged from approximately 110 to 640, with the baseline estimate of 535 at rVE = 25%.

**Figure 3:**
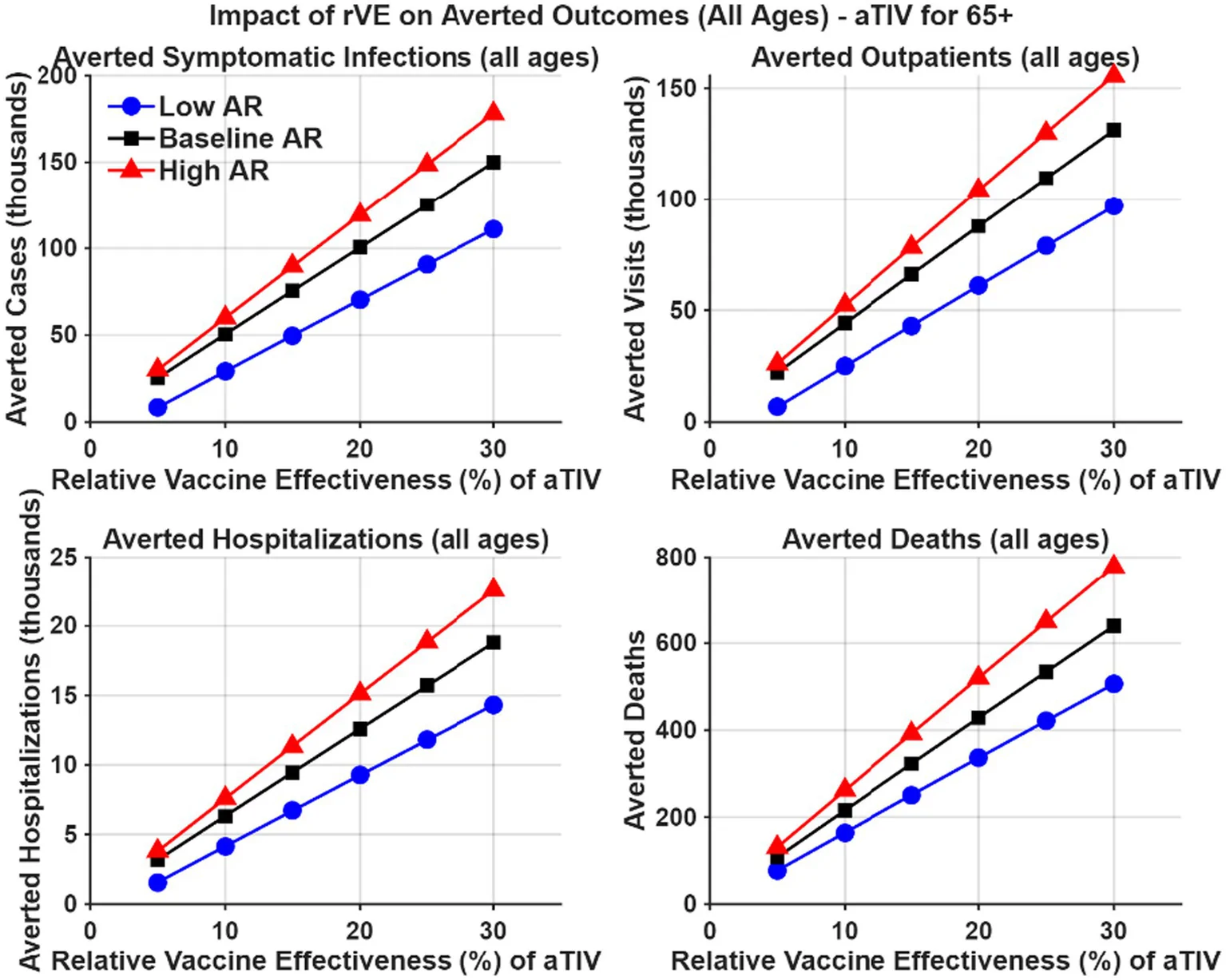
Impact of relative vaccine effectiveness (rVE) on averted outcomes across all ages under three transmission scenarios. Low attack rates (6.4% of population symptomatically infected), baseline (8.0%), and high (9.6%) attack rates are considered.

The impact of transmission intensity on total healthcare costs was evaluated by varying the baseline transmission rate by ±3 and ±6% (Figure 4). For Strategy II, total costs ranged from approximately $1,078 million under the lowest transmission scenario (−6%) to $1,590 million under the highest (+6%). The incremental cost of aTIV relative to QIV decreased as transmission intensity increased, from approximately $34.6 million under low transmission (−6%) to $23.2 million at baseline and less than $10.9 million under the highest scenario (+6%). This convergence occurs because higher transmission intensity amplifies the treatment cost savings attributable to aTIV’s superior effectiveness, progressively offsetting the vaccine price premium. During severe influenza seasons—when disease burden and healthcare costs are greatest, aTIV approaches cost neutrality while simultaneously delivering the largest absolute health benefits in averted hospitalizations and deaths.

**Figure 4:**
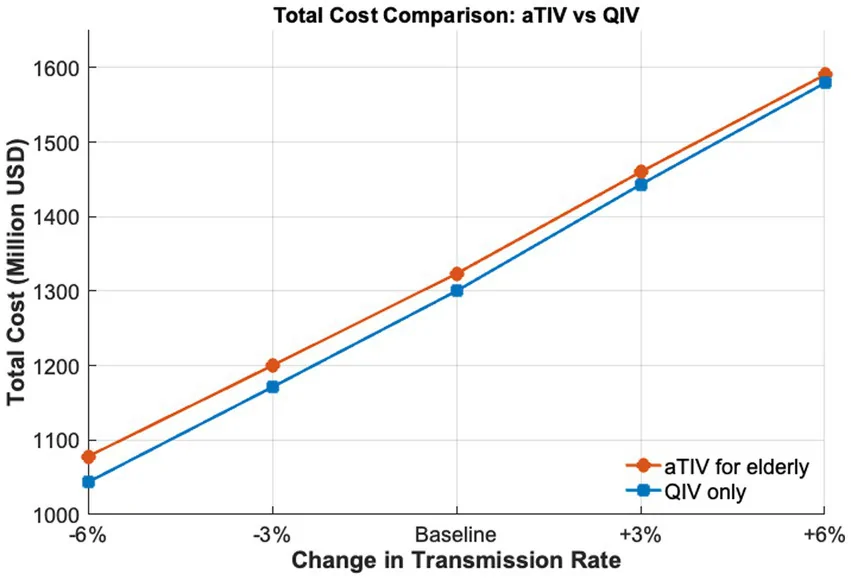
Sensitivity of total healthcare costs to changes in transmission rate under Strategy II (aTIV for adults aged ≥65 years) compared with the QIV-only comparator. Transmission intensity was varied from −6 to +6% relative to the baseline scenario. Total costs include both vaccination program expenditure and influenza-related treatment costs.

To identify the threshold rVE at which aTIV becomes cost-saving, we conducted a break-even analysis under baseline transmission assumptions (Figure 5). Total costs under Strategy II decreased linearly with increasing rVE, from approximately $1,348 million at rVE = 5% to $1,293 million at rVE = 50%. The QIV-only comparator maintained constant costs of $1,300 million across all rVE values, as rVE varies only the aTIV strategy while leaving baseline QIV outcomes unchanged. The break-even point occurred at rVE = 44%, beyond which aTIV adoption becomes both clinically beneficial and cost-saving from the healthcare payer perspective.

**Figure 5:**
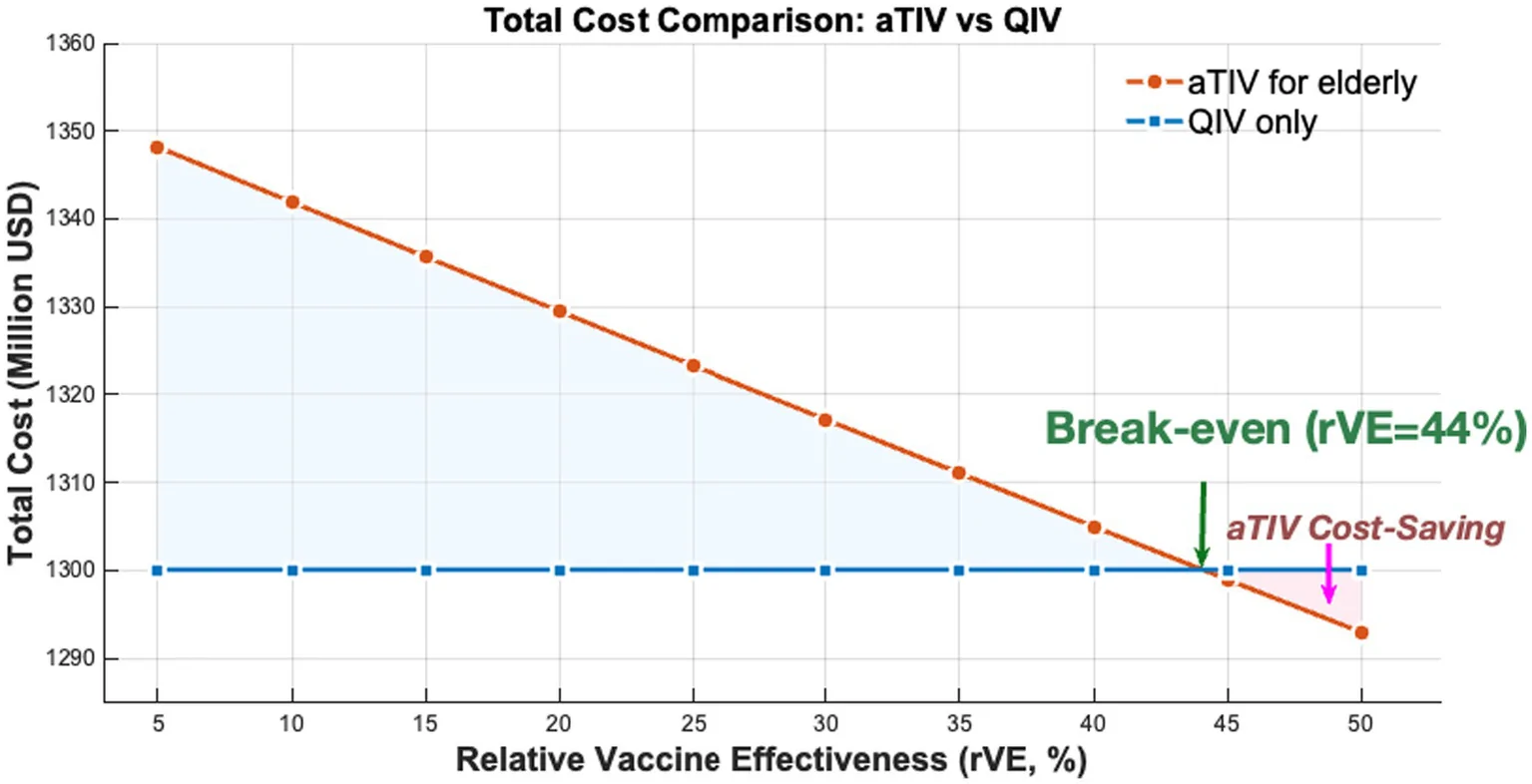
Sensitivity analysis of relative vaccine effectiveness (rVE) for Strategy II. Total healthcare costs under aTIV provision for adults aged ≥65 years (orange line) are compared with the QIV-only comparator (blue dashed line). The break-even point occurs at rVE = 44%, beyond which aTIV becomes cost-saving. The shaded pink region indicates the cost-saving zone where aTIV total costs fall below those of the comparator strategy.

We further conducted a probabilistic sensitivity analysis (PSA) with 1,000 Monte Carlo iterations to quantify joint parameter uncertainty. PSA medians of averted outcomes closely matched the base-case deterministic estimates, with substantial credible intervals reflecting uncertainty in rVE, QIV effectiveness, and costs (Table 4). Strategy II consistently dominated Strategy I across iterations in absolute averted outcomes. Net incremental cost was cost-saving in 15.7% of iterations for Strategy II and 12.3% for Strategy I, indicating that aTIV can yield net savings under favorable parameter combinations such as high rVE or lower aTIV price (Supplementary Figure 1).

## 4 Discussion

This study provides the first dynamic transmission model-based economic analysis of MF59-adjuvanted trivalent influenza vaccine introduction into the Korean National Immunization Program for adults aged ≥65 years, capturing both direct protection effects and herd immunity. Despite historically high vaccination coverage of approximately 80% among adults aged 65 years and older, among the highest globally, Korea continues to experience substantial influenza-related burden in this population, with our model estimating over 3,200 deaths and 84,000 hospitalizations annually. Recent data indicate coverage has declined to approximately 75% in the 2025–2026 season (40), suggesting that maintaining high coverage levels presents ongoing challenges. Combining sustained coverage efforts with improved vaccine effectiveness offers a valuable approach to reducing this disease burden.

A central question for policymakers is whether to implement targeted (Strategy I: aTIV for ≥75 years) or universal (Strategy II: aTIV for ≥65 years) approaches. Strategy I requires approximately half the incremental investment but delivers proportionally smaller health benefits, preventing only 45% as many deaths and 42% as many hospitalizations. Notably, despite including the lower-risk 65–74 year cohort, Strategy II achieves superior efficiency with a lower NNV to prevent one death (15,028 vs. 15,230). This advantage reflects the nonlinear dynamics of infectious disease transmission, as broader coverage amplifies indirect protection, with 26.0% of averted infections under Strategy II occurring among individuals who did not receive aTIV. Based on these findings, Strategy I should be considered a transitional step when budget constraints preclude immediate universal adoption.

From an economic perspective, treatment cost savings offset 57.4% of incremental vaccine costs under Strategy II, a figure that does not achieve cost neutrality but represents substantial value. The net investment of $23.2 million prevents 535 deaths annually, corresponding to approximately $43,000 per death averted. This figure is reported descriptively and is not compared against any formal cost-effectiveness threshold. These figures compare favorably with other publicly funded preventive health interventions and represent a modest 1.8% increase in total influenza-related healthcare expenditure.

A critical question for NIP decision-making is whether the benefits of aTIV are robust under parameter uncertainty. Sensitivity analyses examining rVE values from 5 to 50% demonstrated that health benefits scale substantially with vaccine effectiveness. Our break-even analysis revealed that aTIV becomes cost-saving at rVE = 44%, above central meta-analytic estimates of 10–25% (10, 11, 39), but not implausible given the heterogeneity observed across influenza seasons and the potential for underestimation of aTIV benefits in observational studies subject to residual confounding. Even under the most conservative assumption (rVE = 5%), Strategy II prevents approximately 110 deaths and 3,200 hospitalizations annually, benefits that remain clinically meaningful and justify consideration of aTIV adoption.

Our transmission intensity analyses revealed that benefits scale disproportionately under high transmission scenarios, with important implications for program evaluation. A single-season assessment during a mild influenza year could substantially underestimate the long-term value of aTIV. The incremental cost of aTIV relative to QIV decreased substantially as transmission intensity increased, from approximately $34.5 million under low transmission (−6%) to less than $11.0 million under high transmission (+6%) scenarios. This convergence toward cost neutrality during severe seasons reflects the amplified treatment cost savings achieved through aTIV’s superior effectiveness when disease burden is greatest. H3N2-dominant seasons, historically responsible for the greatest mortality burden in Korea (45), would demonstrate larger benefits. These findings suggest that aTIV represents particularly strong value during severe epidemic years, precisely when enhanced protection is most needed.

The superior efficiency of Strategy II aligns with international trends, as the United Kingdom, Australia, and Italy have incorporated enhanced influenza vaccines into their publicly funded programs for older adults, while the United States and Canada have issued preferential recommendations (13–15, 46–49). Given Korea’s rapidly aging population, with adults aged ≥65 years projected to exceed 40% by 2050 (24), enhanced vaccination strategies will become increasingly important for healthcare system sustainability.

Several limitations should be acknowledged. Our single-season modeling horizon, while appropriate for short-term budget planning and aligned with annual NIP procurement decision cycles, omits demographic processes such as births, background mortality, and aging that influence multi-year disease dynamics. The within-season indirect protection effects we estimate reflect transmission reductions over a single influenza season and should not be extrapolated linearly to multi-year horizons, as inter-seasonal cohort turnover, waning immunity, and antigenic drift would attenuate sustained herd-immunity effects. A multi-season dynamic analysis with explicit demographic structure would be required to evaluate the cumulative population-level impact of aTIV adoption.

Our model treats vaccine-induced protection as homogeneous across infection states, with a single effectiveness parameter reducing both susceptibility to infection and progression to severe outcomes. In reality, influenza vaccines tend to be more effective at preventing hospitalization and death than infection or onward transmission. A more refined leaky model with separate effectiveness parameters against infection, symptomatic disease, and hospitalization would better capture this, but the required empirical inputs are not currently available for the Korean setting. Accordingly, the proportion of averted outcomes attributable to indirect (herd) effects may be somewhat overestimated.

Our model also omits the 10–14-day delay between vaccination and onset of protection. Since most vaccination in Korea is administered in September and October prior to the December to February transmission peak, this ramp-up overlaps with the low-transmission shoulder and is unlikely to substantially affect our conclusions.

The break-even threshold (rVE = 44%) exceeds central estimates from published meta-analyses (10–25%) (10, 11, 39), so achieving cost-saving status may be challenging in an average influenza season under current effectiveness assumptions. However, real-world effectiveness may exceed trial-based estimates during severe seasons, thus the cost-saving threshold should be considered achievable but uncertain.

In summary, both aTIV strategies incur higher total costs than the QIV program but generate substantial reductions in severe outcomes. Strategy II offers greater health impact at a higher incremental cost, while Strategy I provides a more fiscally conservative option with more limited benefits. Strategy II demonstrates superior efficiency with a lower NNV to prevent one death (15,028 vs. 15,230) despite including adults aged 65–74 years with lower baseline mortality risk. Sensitivity analyses confirmed that meaningful health benefits persist even under the most conservative parameter assumptions.

## 5 Conclusion

Introduction of aTIV into the Korean NIP is budget-feasible, with net increases of only 0.9–1.8% in total influenza-related healthcare expenditure. Treatment cost savings offset over half of the incremental vaccine investment, and the modest additional spending delivers substantial reductions in hospitalizations and mortality. Strategy II (universal aTIV for ≥65 years) offers superior value for money and is the preferred approach when budget permits, while Strategy I (targeted aTIV for ≥75 years) provides a fiscally conservative entry point. These findings support aTIV adoption as an economically justified strategy to enhance protection of Korea’s rapidly aging population.

## Data Availability

The original contributions presented in the study are included in the article/Supplementary material, further inquiries can be directed to the corresponding author.
