# Supplementary material for: Economic analysis of adjuvanted trivalent influenza vaccine for older adults in the Korean National Immunization Program
Source: Front Public Health. 2026 Oct 8;14:1833971. doi: 10.3389/fpubh.2026.1833971 (PMC13687060; doi:10.3389/fpubh.2026.1833971)
Supplement: Supplementary file 1 [file Supplementary_file_1.docx]

Appendix Figure S1. Probabilistic sensitivity analysis (PSA) results comparing Strategy I (aTIV for ≥75 years; green) and Strategy II (aTIV for ≥65 years; orange) across 1,000 Monte Carlo iterations. Top row: distributions of averted hospitalizations, averted deaths, and net incremental cost. Bottom row: net incremental cost versus averted deaths, versus rVE, and a box-plot of averted deaths by strategy. Dashed lines mark the cost-saving threshold (net incremental cost = 0). PSA sampled rVE, age-specific QIV vaccine effectiveness, treatment unit costs, and the aTIV price multiplier (Section 3.7).
